# Severe conjunctival allergic reaction to polyglactin 910 sutures following strabismus surgery: A case report

**DOI:** 10.1016/j.ijscr.2024.110168

**Published:** 2024-08-13

**Authors:** Khalid S. AlOtaibi, Rafaa Babgi, Gorka Sesma

**Affiliations:** Pediatric Ophthalmology and Strabismus Division, King Khaled Eye Specialist Hospital, Riyadh, Saudi Arabia

**Keywords:** Strabismus surgery, Polyglactin 910 (Vicryl) sutures, Conjunctival allergic reaction, Suture removal, Postoperative complications

## Abstract

**Introduction and importance:**

Polyglactin 910 sutures are commonly used in strabismus surgery due to their favorable handling and absorption qualities. However, their potential to cause allergic reactions is poorly documented in medical literature. This case report emphasizes this rare complication, stressing the importance of promptly recognizing and managing such reactions to ensure optimal patient outcomes.

**Case presentation:**

An 11-year-old girl with a history of left congenital partial third nerve palsy was treated with 8–0, polyglactin 910 sutures during a strabismus surgery. However, two days postoperatively, she experienced persistent redness, swelling, and pain in her left eye despite antibiotic therapy. After six days, the sutures were removed, resulting in an immediate reduction in symptoms. By the two-month follow-up, the patient fully recovered, with no signs of inflammation or complications.

**Clinical discussion:**

This case highlights a rare but crucial allergic reaction to polyglactin 910 sutures used in strabismus surgery. The patient's persistent inflammation, pain, and resistance to antibiotics indicated a localized allergic reaction, rather than an infection. The prompt resolution of symptoms after suture removal supports the diagnosis of suture-related allergic reaction. This case emphasizes the need to consider suture material as a potential cause of postoperative complications, especially when standard treatment fails.

**Conclusion:**

Healthcare providers should be alert to potential allergic reactions to sutures after strabismus surgery. Timely identification and removal are vital for resolving the symptoms and achieving optimal patient outcomes. This case emphasizes the importance of postoperative care protocols that consider allergies to suture material.

## Highlights

• After the strabismus surgery, a severe conjunctival allergic reaction occurred with polyglactin 910 sutures.
• The removal of the suture resulted in a significant improvement within 24 hours.
• Emphasizes the importance of considering suture-related allergies in assessing postoperative complications.
• Stresses the necessity of quickly identifying and treating allergic reactions related to sutures.
• Offers a comprehensive review of allergy management in pediatric strabismus surgery.

## 1 Introduction

Strabismus surgery, a common ophthalmic procedure, corrects eye misalignment in both children and adults. Polyglactin 910 (Vicryl, Ethicon Inc., Somerville, NJ, USA) is a synthetic suture commonly used in these operations, due to its absorbable nature and minimal need for removal, with complete absorption in 56–70 days [[1], [2], [3]].

Although polyglactin 910 offers benefits, some patients may develop allergic reactions. Research indicated a severe eye inflammation after strabismus surgery, which resolved after removing sutures and administering topical cyclosporine and steroids [4,5].

Current literature lacks detailed information on allergic reactions to polyglactin 910 sutures in pediatric strabismus surgery. Studies have explored general outcomes and complications, but few focus on suture-related allergic reactions in children [6,7]. Suture removal's effectiveness and optimal management remain unknown. Anecdotal evidence suggests relief after removal, but systematic studies are lacking [8].

This case report aimed to detail a severe allergic reaction to 8-0, polyglactin 910 sutures in a pediatric patient, highlighting the importance of accurate identification and treatment. Our goal is to provide valuable insights and enhance clinical practice by thoroughly analyzing this rare complication and the management techniques employed.

## 2 Presentation of case

The present case report has been organized and presented in accordance with the SCARE (Surgical CAse REport) criteria, with the aim of meeting the highest standards for reporting surgical case studies [9].

An 11-year-old girl with a history of left congenital partial third nerve palsy presented to the pediatric clinic at King Khaled Eye Specialist Hospital for evaluation and management of strabismus.

After consulting the pediatrician's report on the patient's magnetic resonance imaging (MRI) results, we verified that the cause of the cranial nerve III palsy in this case was perinatal hypoxia. The MRI findings showed evidence of hypoxic-ischemic damage to the midbrain region, specifically targeting the oculomotor nerve nucleus and/or its associated fibers.

It is crucial to highlight that the patient does not exhibit any other long-term effects from the perinatal hypoxic episode. The isolated nature of the cranial nerve III palsy, without additional neurological deficits, suggests a localized impact of the hypoxic injury.

Although we do not possess immediate access to the MRI images, the radiological interpretation furnished by the pediatrician provides robust support for this etiological conclusion. The correspondence between the clinical findings and the imaging results reinforces our diagnostic appraisal.

The visual acuity was found to be 20/20 in the right eye and 20/40 in the left eye, with a refractive power of +1.00 diopters in both eyes. Extraocular motility examination demonstrated full motion in the right eye, while the left eye showed marked limitation in supraduction and adduction, with a value of -3. Orthoptic workup revealed left exotropia of 70 prism diopters (PD), left hypotropia of 20 PD as the primary deviation, and right 100 PD exotropia and 40 PD hypertropia as secondary deviations. A measurement of 400 s of arc was obtained in the stereopsis assessment. Slit-lamp examination was unremarkable, with a quiet conjunctiva, clear cornea, deep and quiet anterior chamber, and a healthy optic disc with a flat retina.

The patient's clinical course began with strabismus surgery. General anesthesia was administered for left eye lateral rectus recession (9.0 mm) and inferior rectus recession (7.0 mm) through a fornix-based approach in the inferotemporal region with the hang-back technique and 6-0, polyglactin 910 sutures for the muscle and 8-0, polyglactin 910 sutures for conjunctival closure. Following surgery, we prescribed neomycin sulfate, polymyxin B sulfate, dexamethasone drops, and an ointment (Maxitrol, Alcon Labs, Inc. Fort Worth, TX, USA).

**Fig. 1:**
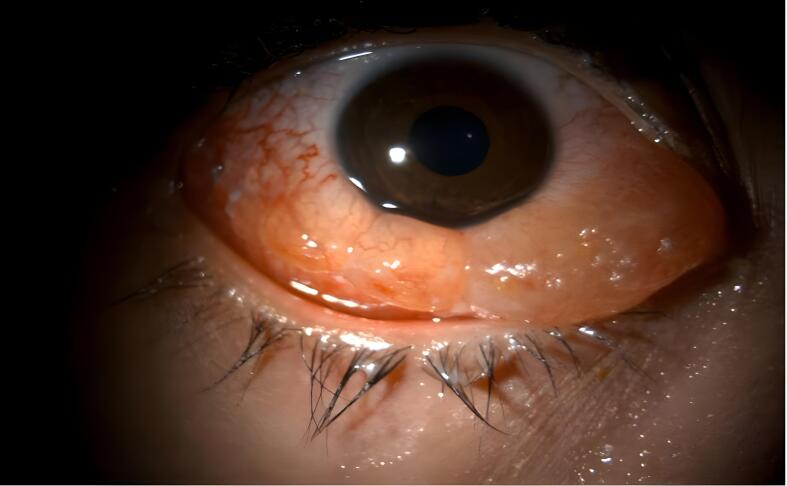
Slit lamp photograph. Conjunctival hyperemia and chemosis in a pediatric patient post-strabismus surgery. The image featured in the text illustrates the left eye of a pediatric patient exhibiting significant conjunctival hyperemia and chemosis. Inflammation in the conjunctiva suggests an inflammatory response. The patient's clinical presentation raises the possibility of an allergic reaction, potentially to suture materials such as polyglactin 910 or an infectious process. Although no signs are commonly associated with infection, the presence of pain warrants the consideration of an infection control protocol. This case highlights distinguishing between allergic and infectious etiologies of postoperative ocular inflammation to ensure appropriate management and treatment.

**Fig. 2:**
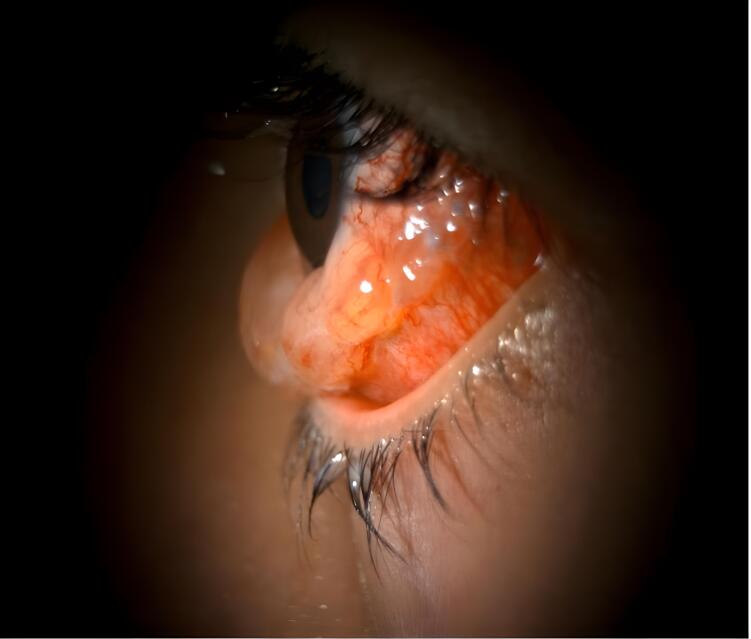
Slit lamp photographs. Pronounced conjunctival hyperemia and chemosis post-strabismus surgery. The accompanying image depicts a pediatric patient who experienced severe conjunctival hyperemia and chemosis following strabismus surgery. Significant redness and swelling of the conjunctiva suggested an acute inflammatory response. This presentation could be due to an allergic reaction, potentially to the suture material (polyglactin 910), or an infectious etiology. Even though the clinical signs leaned toward an allergic reaction, an infection control protocol was initiated owing to the presence of pain.

Two days after the surgery, the patient sought treatment at the Emergency Department due to redness, swelling of the eyelid, and severe pain in the left eye, which made it difficult for them to sleep. The patient had mild conjunctival inflammation and significant swelling, which prevented eyelid closure and caused discharge on the conjunctival sutures. Normal examination results were found, with no signs of inflammation or effects on intraocular structures or ocular movements (Fig. 1, Fig. 2). The individual in question exhibited a residual exotropia of 15 PD, -3 medial rectus function with the cosmetic outcome deemed acceptable by the patient.

The patient's presentation included signs and symptoms suggestive of an allergic reaction, however, the presence of pain and discharge over the sutures made it difficult to rule out an underlying infection. To ensure comprehensive care and prevent potential complications, we initiated an infection control protocol. The patient was admitted, and a conjunctival swab was collected for culture. Topical Moxifloxacin (Vigamox 0.5 %, Alcon Labs, Inc. Fort Worth, TX, USA) administered every 8 h, and intravenous antibiotics, including gentamycin (dosed at 5 mg/kg/day, administered in three equal doses every 8 h) and Cefazoline Sodium (dosed at 50 mg/kg/day given in three doses every 8 h) were initiated.

After the patient's health did not improve with antibiotic therapy for two days, we replaced the initial therapy with intravenous ceftriaxone, vancomycin and add 1 % topical prednisolone acetate to address ongoing inflammation. Conjunctival swab cultures remained negative throughout the treatment. Despite administering the new antibiotic therapy for persistent symptoms and clinical findings, we concluded that removing the conjunctival sutures on the sixth day following surgery was necessary. This procedure was carried out on the same day.

**Fig. 3:**
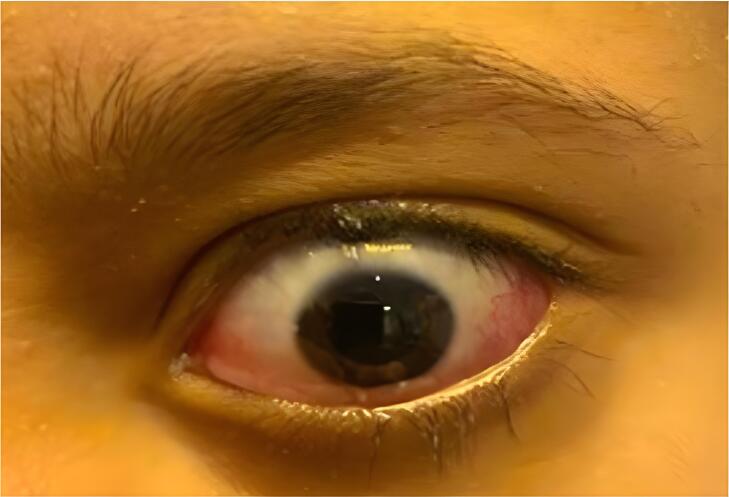
Conjunctival injection improvement in the left eye post-suture removal. The image illustrates conjunctival injection improvement and disappearance of chemosis in the left eye of a patient, predominantly in the inferior quadrant, following the removal of suture material compared to prior observations. This suggests that the initial symptoms were likely due to a foreign body reaction or allergic response to the sutures. Conjunctival swelling was markedly reduced, and hyperemia was less severe. The periorbital skin, pupils, and eyelids were normal. This presentation emphasizes the importance of considering suture-related complications in postoperative ocular inflammation and highlights the effectiveness of suture removal in resolving these symptoms.

**Fig. 4:**
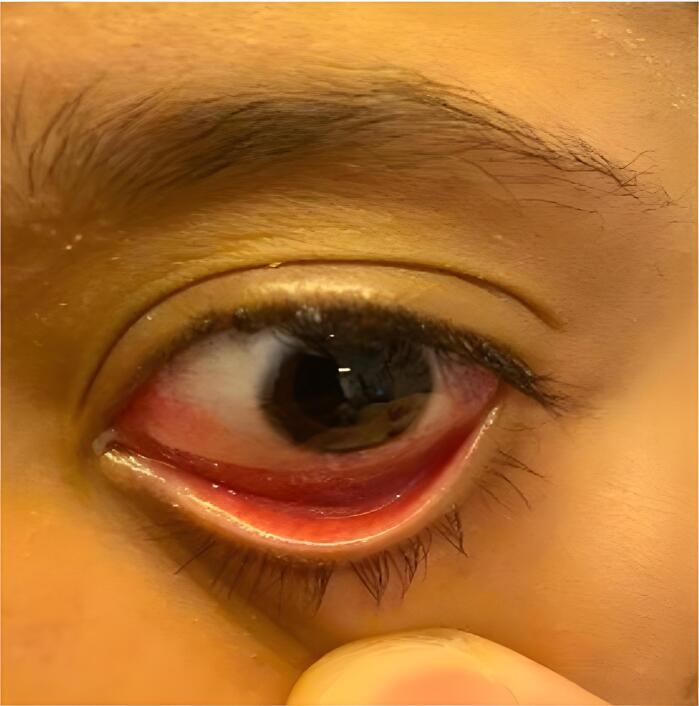
Conjunctival injection improvement in the left eye post-suture removal. The photograph shows a significant improvement in the conjunctival injection of the left eye of a patient, particularly in the inferior fornix, after suture removal for 24 h. The image depicts a noticeable reduction in chemosis compared to the previous photos, which suggests a positive response to removing the allergenic suture. However, persistent conjunctival injection indicates an ongoing improvement in inflammation, possibly due to residual allergic conjunctivitis or mild irritative conjunctivitis.

Within 24 h of suture removal, the patient exhibited substantial improvement in swelling, redness, and disappearance of pain (Fig. 3, Fig. 4). The patient's response to the treatment allowed for discharge on the eighth postoperative day. Significant improvement was noted after suture removal, following adjustments to the treatment regimen.

After a two-month follow-up appointment, the patient's left eye showed no signs of redness, inflammation, or discomfort, and the symptoms were fully resolved after suture removal. The patient's adherence to the prescribed antibiotic regimen was confirmed, and no adverse events were reported following suture removal.

## 3 Discussion

This report presents the case of an 11-year-old female patient who experienced severe conjunctival hyperemia and chemosis after undergoing strabismus surgery. The patient's symptoms, including redness, swelling, and pain, did not improve after the antibiotic therapy. However, they were significantly alleviated after removal of the conjunctival 8-0, polyglactin 910 sutures. Postoperative complications may require consideration of suture-related allergic reactions and suture removal as a management strategy.

Polyglactin 910, a copolymer of 90 % glycolide and 10 % l-lactide, is a widely used synthetic absorbable suture material in various surgical procedures, including ophthalmic surgeries [2]. However, it has been associated with allergic reactions in some patients, and the underlying mechanisms are not yet clear, but are thought to involve immunological and inflammatory processes [5].

The immune system reacts to foreign substances by activating an inflammatory response, which includes the release of histamines and other molecules [5].

Polyglactin 910 generally elicits type IV hypersensitivity reactions, also referred to as delayed-type hypersensitivity reactions [10]. Sensitized T cells activated by specific antigens such as suture material or its degradation products initiate these reactions. Upon subsequent exposure to antigens, cytokines are secreted that attract and activate macrophages, leading to an inflammatory response and granuloma formation, characterized by the accumulation of macrophages, lymphocytes, and giant cells near the suture material [11].

However, while the majority of suture reactions typically belong to the Type IV classification, the instantaneous onset observed in our case (48 h) seems to indicate a potential inclusion of Type I mechanism. Delves et al. (2017) offer an extensive examination of hypersensitivity responses, which may offer valuable insights into the immunological processes that could be pertinent to our case [12].

Polyglactin 910 degradation results in various breakdown products, including glycolic and lactic acids, which can act as haptens when bound to carrier proteins, potentially triggering an immune response and allergic reactions [13,14]. Katz et al. (2005) delved into the possibility of suture materials inducing allergic reactions, although their examination primarily centered on non-absorbable sutures [15].

Polyglactin 910 sutures absorb proteins, including fibrinogen, albumin, and immunoglobulins, from surrounding tissue fluids [16]. These absorbed proteins may change the surface properties of the suture material, making it more likely to cause an immune response [17]. This process can lead to an allergic reaction to suture materials.

Bacterial contamination may exacerbate allergic reactions to polyglactin 910 sutures by promoting the formation of biofilms, which shield bacteria from the host's immune system and antibiotics. This can lead to persistent infections and inflammation, worsening the inflammatory response and contributing to suture-related allergic reactions [18,19].

Individual genetic factors may affect their susceptibility to allergic reactions caused by polyglactin 910 sutures. Variations in genes responsible for immune regulation, including cytokine and T-cell receptor genes, can influence the severity and duration of the inflammatory response to the suture material [20].

Polyglactin 910 sutures are more likely to cause allergic reactions in the conjunctiva, which has a higher concentration of immune cells like mast and dendritic cells, responsible for allergic responses, compared to muscle tissue [21]. Rapid immune cell recruitment, due to environmental allergens and a large, well-vascularized surface area, triggers allergic reactions.

This research supports previous findings on allergic reactions to suture materials used in ophthalmic surgeries. Demir et al., Chung et al. reported severe conjunctival allergic reaction due to polyglactin 910 sutures. These resolved after suture removal [5,22]. This underscores the significance of recognizing this possible complication among ophthalmologists.

Our patient's rapid improvement after suture removal sets this case apart from others. Unlike previous cases, which documented symptom resolution weeks after surgery, our patient experienced significant improvement within 24 h of suture removal and complete resolution by the two-month follow-up [5,22]. Variations in the response might be due to the severity of the allergic reaction, the timing of suture removal, or individual patient characteristics.

While the clinical presentation and the quick resolution following suture removal are strong indicators of an allergic reaction, histopathological evidence would offer a more definitive confirmation. Conjunctival scrapings for cytology, as noted by Leonardi et al. (2012), can be a valuable tool in diagnosing allergic conjunctivitis, with eosinophils being a key indicator [23].

In future cases, the use of conjunctival scraping or biopsy for cytology and histopathology could prove to be valuable diagnostic tools.

In situations where a suture removal is not possible or advisable due to a suspected allergy, alternative medical interventions can be considered. A study conducted by Bilkhu et al. (2019) evaluated the effectiveness of various treatments for allergic conjunctivitis, such as antihistamines and corticosteroids [24]. Nevertheless, it is essential to recognize that the use of these treatments should not postpone the suture removal process if the reaction is severe or persists.

The case report showcases a patient's clinical course in detail, supported by a complete resolution over a long-term follow-up. Additionally, it includes a comprehensive literature review that provides context to the findings. The major limitation, however, is that it is a single case report, which may not be universally applicable to all patients with suture-related allergic reactions.

Our case presentation underscores the critical aspects of post-strabismus surgery complications in pediatric patients. First and foremost, the early onset of severe symptoms, appearing just two days post-operation, emphasizes the necessity for vigilant monitoring during the immediate postoperative period. Secondly, the presentation mimicked an infectious etiology, creating a diagnostic challenge that led to unnecessary intravenous antibiotic courses. This emphasizes the need to consider non-infectious causes, particularly allergic reactions to suture materials, in the differential diagnosis of postoperative complications. Finally, the rapid and complete symptom resolution within 24 h after suture removal, sustained at the two-month follow-up, suggests suture removal as a therapeutic option for suspected allergic reactions. These points collectively highlight the importance of clinician awareness regarding rare but significant complications like suture allergies, potentially improving patient outcomes and reducing unnecessary interventions in pediatric ophthalmology.

Close postoperative monitoring and a high level of suspicion for complications resulting from sutures are crucial for patients who have had strabismus surgery. Ophthalmologists should promptly diagnose and manage allergic reactions, considering suture removal as a therapeutic option. Patient education about postoperative symptoms and prompt follow-up appointments is crucial.

Recognizing the factors that contribute to the risk of allergic reactions in pediatric patients undergoing strabismus surgery is crucial for future research. Prospective studies utilizing various suture materials and techniques may help to minimize these risks. Immunological mechanisms may aid in developing targeted prevention and treatment strategies.

## 4 Conclusion

Polyglactin 910 sutures can lead to allergic reactions resulting from immunological responses, biochemical processes, and bacterial contamination. Managing these reactions involves comprehending their mechanisms and creating novel suture materials. Postoperative care and prompt intervention are crucial for patient outcomes. Future research should focus on understanding the incidence, risk factors, and mechanisms of suture-related allergic reactions to develop guidelines for prevention and management.

## 5 Patient perspective

The patient and her family's initial concerns regarding the severity of the postoperative reaction were alleviated upon discovering that suture removal resulted in a swift improvement in symptoms. They expressed satisfaction with the care they received and the outcomes of the intervention, underscoring the significance of close follow-up and prompt management of postoperative complications.

## Informed consent

Written informed consent was obtained from the patient's parents/legal guardian for publication and any accompanying images. A copy of the written consent is available for review by the Editor-in-Chief of this journal on request. The Institutional Review Board of King Khaled Eye Specialist Hospital approved this study (identification number RP 23021-CR). This case report was conducted in accordance with the principles outlined in the Declaration of Helsinki, which guarantees ethical standards and protects patient rights.

## Ethical approval

This study received ethical approval from the Institutional Review Board (IRB) of King Khaled Eye Specialist Hospital (KKESH) under the approval number RP 23021-CR. The study was conducted in accordance with the principles outlined in the Declaration of Helsinki, ensuring adherence to ethical standards and the protection of patient rights. Informed consent for publication of this case report was obtained from both parents of the patient.

## Financial support

None.

## CRediT authorship contribution statement

The authors made the following contributions to this work:

Conceptualization: RB, GS.

Methodology: KA, RB, GS.

Software: KA, GS.

Validation: KA, RB, GS.

Formal Analysis: KA, RB, GS.

Investigation: KA, GS.

Resources: KA, RB.

Data Curation: KA, RB, GS.

Writing – Original Draft: KA.

Writing – Review & Editing: KA, RB, GS.

Visualization: RB, GS.

Supervision: GS.

Project Administration: RB, GS.

Funding Acquisition: GS.

Each author has approved the final version of the manuscript and agrees to be accountable for all aspects of the work, ensuring that questions related to the accuracy or integrity of any part of the work are appropriately investigated and resolved. Khalid S AlOtaibi and Rafaa Babgi contributed equally to this work and should be considered co-first authors.

## Guarantor

Gorka Sesma.

## Declaration of Generative AI and AI-assisted technologies in the writing process

During the preparation of this work, the author(s) used Claude 3, an AI language model developed by Anthropic PBC, was used to enhance grammar, coherence, and readability. After using this tool/service, the authors reviewed and edited the content as required and took full responsibility for the content of the publication.

## Declaration of competing interest

The authors declare that there are no conflicts of interest regarding the publication of this case report.
